# Evolution of Reproductive Behavior

**DOI:** 10.1534/genetics.119.302263

**Published:** 2019-12-31

**Authors:** Robert R. H. Anholt, Patrick O’Grady, Mariana F. Wolfner, Susan T. Harbison

**Affiliations:** * Center for Human Genetics, Clemson University, Greenwood, South Carolina 29646; ^†^ Department of Genetics and Biochemistry, Clemson University, Greenwood, South Carolina 29646; ^‡^ Department of Entomology, Cornell University, Ithaca, New York 14853; ^§^ Department of Molecular Biology and Genetics, Cornell University, Ithaca, New York 14853; ** Laboratory of Systems Genetics, National Heart Lung and Blood Institute, National Institutes of Health, Bethesda, Maryland 20892

**Keywords:** *Drosophila*, adaptation, genetics, chemoreception, multigene family, gene–environment interaction, natural variation, selection, mating, courtship, song, postmating behaviors, fitness, pheromones, wing spots, seminal proteins, FlyBook

## Abstract

Behaviors associated with reproduction are major contributors to the evolutionary success of organisms and are subject to many evolutionary forces, including natural and sexual selection, and sexual conflict. Successful reproduction involves a range of behaviors, from finding an appropriate mate, courting, and copulation, to the successful production and (in oviparous animals) deposition of eggs following mating. As a consequence, behaviors and genes associated with reproduction are often under strong selection and evolve rapidly. Courtship rituals in flies follow a multimodal pattern, mediated through visual, chemical, tactile, and auditory signals. Premating behaviors allow males and females to assess the species identity, reproductive state, and condition of their partners. Conflicts between the “interests” of individual males, and/or between the reproductive strategies of males and females, often drive the evolution of reproductive behaviors. For example, seminal proteins transmitted by males often show evidence of rapid evolution, mediated by positive selection. Postmating behaviors, including the selection of oviposition sites, are highly variable and *Drosophila* species span the spectrum from generalists to obligate specialists. Chemical recognition features prominently in adaptation to host plants for feeding and oviposition. Selection acting on variation in pre-, peri-, and postmating behaviors can lead to reproductive isolation and incipient speciation. Response to selection at the genetic level can include the expansion of gene families, such as those for detecting pheromonal cues for mating, or changes in the expression of genes leading to visual cues such as wing spots that are assessed during mating. Here, we consider the evolution of reproductive behavior in *Drosophila* at two distinct, yet complementary, scales. Some studies take a microevolutionary approach, identifying genes and networks involved in reproduction, and then dissecting the genetics underlying complex behaviors in *D. melanogaster*. Other studies take a macroevolutionary approach, comparing reproductive behaviors across the genus *Drosophila* and how these might correlate with environmental cues. A full synthesis of this field will require unification across these levels.

*DROSOPHILA melanogaster*, as well as other members of the genus *Drosophila*, has served as a model system in genetics, evolution, and development for over 100 years, and as an important model in behavioral studies for nearly as long. Some of the earliest comparative studies were done by A. H. Sturtevant (1915), and examined courtship behaviors and sexual recognition between closely related *Drosophila* species. Subsequent work by Dobzhansky (1946), Spieth (1947), and others further developed *Drosophila* as not only a model for courtship and mating behavior, but as one of the key experimental systems responsible for establishing the biological species concept (Mayr 1982), and our understanding of how species diversify and evolve (Coyne and Orr 1989, 1997).

Behaviors are more than simply the expression of the nervous system; they include complex interactions with various biotic and abiotic factors. Studies on *Drosophila* behavior have benefited from a diverse range of experimental approaches. For example, geneticists correlate gene expression and gene interaction studies with complex behavioral phenotypes. Ecologists elucidate the underlying biotic and abiotic stimuli giving rise to complex behaviors. Evolutionary biologists reconstruct the history of the behavior and the genes underlying those behaviors. A comprehensive approach, incorporating aspects of several disciplines, yields the most robust, and we would argue, biologically relevant and interesting results.

In this article, we focus on the evolution of reproductive behaviors in *Drosophila*. Classical genetic and genomic studies on *D. melanogaster* have had a significant impact on our understanding of behavior through powerful tools that are available to genetically dissect the molecular and neural pathways, and networks that underlie complex behaviors. The genus *Drosophila* is an exceptional model system for understanding the evolution of behavior, including reproductive behaviors. The *melanogaster* species subgroup consists of nine species, divided into four different species complexes. Two of these, the *melanogaster* and *simulans* complexes, are extensively studied models of species formation. The *melanogaster* complex consists of a single species, *D. melanogaster*, and is sister to three sibling species: *D. simulans*, *D. mauritiana*, and *D. sechellia*. There is extensive literature relating to behaviors, in their ecological settings, of multiple species in the genus *Drosophila*, allowing a comparative approach that can help determine how various behaviors may have evolved over a macroevolutionary scale spanning > 60 MY and in response to a diverse set of selection pressures (including, but not limited to, environmental conditions, host plant chemistry, predation pressures, and reproductive isolation). Sequenced genomes exist (Drosophila 12 Genomes Consortium *et al.* 2007; Song *et al.* 2011; Miller *et al.* 2018; Yang *et al.* 2018) for > 20 species, permitting comparative evolutionary studies on the genes that underlie behaviors. At the same time, the genus *Drosophila* contains a major genetic model system, *D. melanogaster*, that has been the subject of study by many laboratories for many behaviors, and their neural and genetic underpinnings, providing a framework from which to consider variation within and between *Drosophila* species. The community has assembled a series of resources that allow evolutionary studies at both the micro- and the macrolevel. Resources for the microlevel include the *Drosophila* Synthetic Population Resource, a population of recombinant inbred lines derived from an advanced intercross population of eight founder strains (King *et al.* 2012; Long *et al.* 2014), and the *D. melanogaster* Genetic Reference Panel (DGRP), a publicly available resource of 205 fully sequenced lines that harnesses naturally occurring variants for the dissection of complex traits, including behaviors (Mackay *et al.* 2012; Huang *et al.* 2014), and can identify candidate genes by association that can be tested genetically. The macrolevel is represented by the existence and study of many species whose phylogenic relationships are known, and for which genomes, natural biology, and descriptive studies are available. Finally, the advent of clustered regularly interspaced short palindromic repeats (CRISPR)/Cas technologies (*e.g.*, Jinek *et al.* 2012; Bassett *et al.* 2013; Gratz *et al.* 2013) allows one to toggle back-and-forth between the behavioral consequences of pathways that are known in *D. melanogaster* and ways that they can be modulated based on results from other species.

Selective forces act on genetic variation within a population. Natural selection can change allele frequencies of mutations that improve or reduce fitness. Thus, a precondition for any behavior to evolve is that there is genetic variation for the behavior within the population. The DGRP and other resources mentioned above have revealed extensive natural variation for behavioral phenotypes (Brown *et al.* 2013; Harbison *et al.* 2013; Swarup *et al.* 2013; Appel *et al.* 2015; Arya *et al.* 2015; Ayroles *et al.* 2015; Garlapow *et al.* 2015; Shorter *et al.* 2015; Carbone *et al.* 2016; Rohde *et al.* 2017; Wu *et al.* 2018a), including reproductive behaviors (Turner *et al.* 2013; Gaertner *et al.* 2015). The genetic architectures underlying behaviors are often complex, allowing very fine-grained effects when selective forces result in the redistribution of allele frequencies leading to modifications of the genetic networks that orchestrate the behavior. Gene-by-gene and gene-by-environment interactions among genes or their networks are prominent hallmarks of behavioral phenotypes (Fedorowicz *et al.* 1998; Sambandan *et al.* 2006, 2008; Rollmann *et al.* 2007, 2008; Yamamoto *et al.* 2008, 2009; Kent *et al.* 2009; Zwarts *et al.* 2011; Huang *et al.* 2012; Swarup *et al.* 2012, 2013; Shorter *et al.* 2015; He *et al.* 2016).

This review leans heavily on the *D. melanogaster* literature for an understanding of the genetic mechanisms underlying specific reproductive behaviors and their variation. We then attempt to integrate this genetic information in a phylogenetic context to discuss how such behaviors may have arisen across the genus *Drosophila*. The obvious caveat here is that there is a large disconnect between what we know in *D. melanogaster* and the diverse behaviors that are observed across the genus. This is because behaviors, particularly those relating to reproduction, are among the most rapidly evolving traits. Accurately and completely reconstructing changes that may have taken place over relatively short time periods millions of years ago is a challenging analytical problem. Here, we present hypotheses for how divergent reproductive behaviors may have evolved, including given the genes known from studies of *D. melanogaster*. We will conclude with a *Perspectives* section summarizing the current understanding in the field and suggesting future studies.

## *Drosophila* Reproductive Behavior

Adult *Drosophila* perform a series of behaviors linked to survival and fitness. Male and female *Drosophila* must locate mating substrates, most of which are also associated with feeding resources. Once in the mating arena, flies must identify conspecifics and, in some cases, compete for access to partners. The latter often encompasses aggressive behaviors to defend feeding and mating opportunities. Once mating has been successful, females must locate a suitable location to oviposit. Both biotic and abiotic stimuli impact reproductive behaviors. For example, social conditions can affect reproduction (Krupp *et al.* 2008).

### Courtship and mating

A diverse array of behaviors in the genus *Drosophila* are associated with reproduction, including those that occur prior to mating, such as male–male aggression and various courtship displays by both males and females, and ones taking place following intromission, such as male guarding and changes in female remating behavior. Many of these behaviors often require specialized morphologies of genitalia, forelegs, mouthparts, and wings; modifications to those structures evolve with changes in those behaviors (Tanaka *et al.* 2009, 2015). Likewise, several molecules are associated with reproductive behaviors, including seminal proteins and pheromones, and these too evolve with the behaviors [Swanson *et al.* (2001) and Haerty *et al.* (2007); reviews include Swanson and Vacquier (2002), and Panhuis *et al.* (2006); the neurogenetics of female *D. melanogaster* reproductive behaviors has been reviewed by Laturney and Billeter (2014)]. Mating and courtship behaviors, as well as associated reproductive traits, tend to evolve rapidly, in part because of their importance in species isolation mechanisms, and in part because of sexual conflicts between males (sperm competition), and between the “interests” and strategies of females and males (Swanson and Vacquier 2002; O’Grady and Markow 2012). These behaviors include aggressive male–male competition, sexual selection acting on a range of characteristics, and sexual antagonism between conspecific males and females resulting in a coevolutionary arms race. The cost of sexual selection was demonstrated by an elegant laboratory evolution study in which single *D. melanogaster* females were mated with single males for 47 generations. Removal of sexual selection over many generations of forced monogamy resulted in decreased intermale aggression and increased resistance of females to male-induced postmating effects (Holland and Rice 1999).

Behaviors, because of their rapid evolution and highly plastic nature, can quickly establish barriers between species. The diversity of reproductive behaviors observed among *Drosophila* species is an important component of this isolation in this genus (Markow and O’Grady 2005, 2006, 2008; O’Grady and Markow 2012). Traditionally, such barriers can arise among premating, mating, postmating prezygotic, and zygotic behaviors, depending on when they occur during mating and reproduction. Premating isolation mechanisms can help individuals identify conspecifics, thus preventing matings between different species. Mating barriers are important, so individuals do not waste valuable resources (*e.g.*, gametes and energy) on nonproductive matings. However, should interspecies matings occur, postmating prezygotic and zygotic barriers can decrease the reproductive success of the cross-species mating.

### Premating and mating

**Figure 1:**
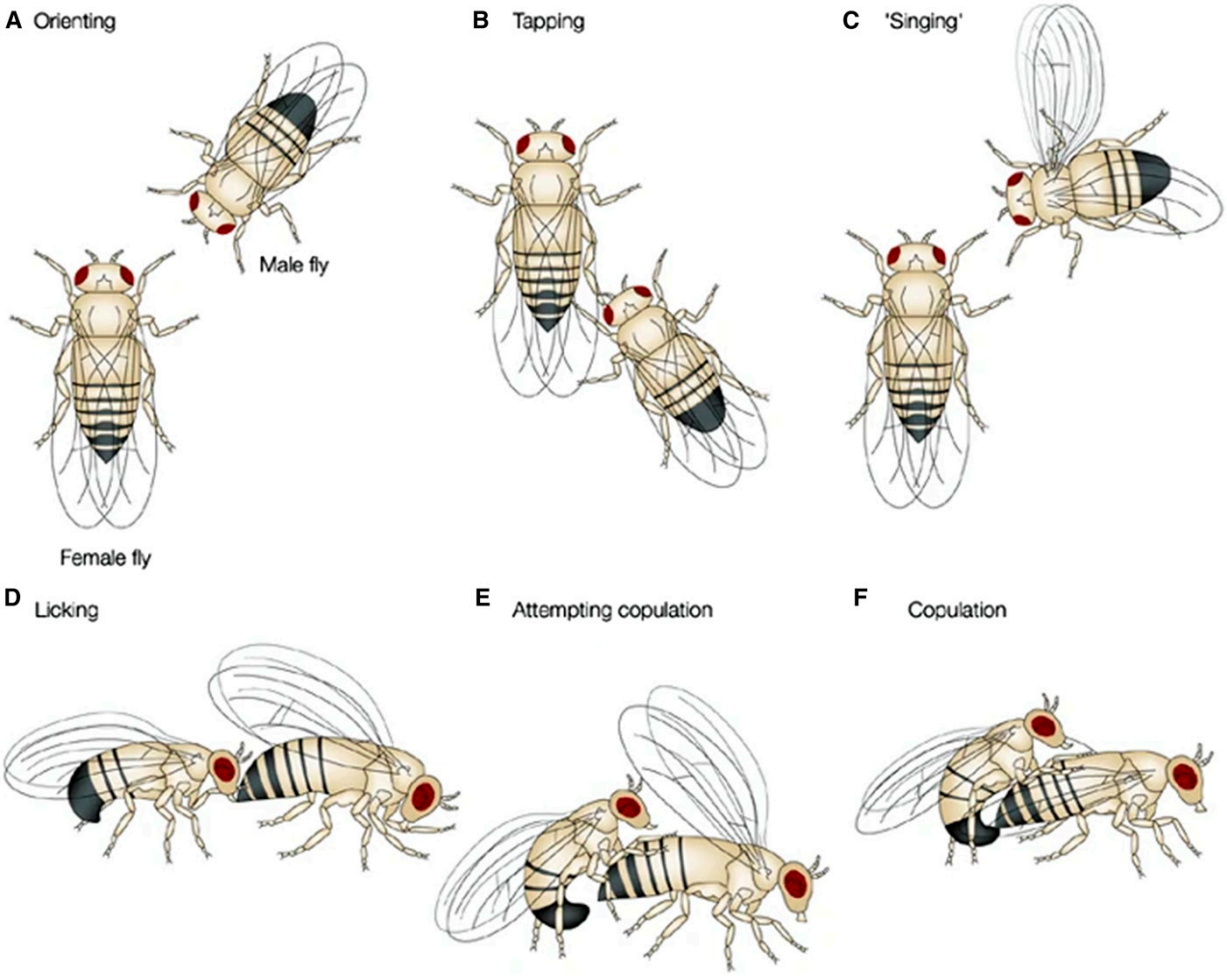
A diagram showing the sequence of behaviors during courtship in *D. melanogaster*. Orienting (a), tapping (b), ‘singing (c), licking (d), attempting copulation (e), and copulation (f). Reprinted, with permission, from Sokolowski (2001).

Premating behaviors allow males and females to recognize and assess partners to determine whether the species, reproductive state, and condition of the partner are appropriate for and conducive to mating. Courtship rituals in *Drosophila* follow a multimodal pattern, mediated through visual, chemical, tactile, and auditory signals, and consist of a sequence of orientation, genital licking, courtship song through wing vibration, and attempted copulation [Hall (1994); reviewed in Sokolowski (2001); Figure 1]. Courtship behaviors include locating a potential mate by using visual or olfactory cues, and communicating with and assessing the potential mate by olfactory, auditory, and visual cues during courtship. Behaviors during mating also are important for reproductive fitness (Markow and O’Grady 2008) and are variable. These include copulation duration as well as interactions that determine whether gametes will be present for fertilization. Although the courtship ritual of *D. melanogaster* males was once considered a defined stereotypical progression of behavioral elements, studies using the DGRP showed extensive heritable variation in courtship patterns (Gaertner *et al.* 2015). Selection acting on variation in any of the sensory inputs that drive the component behaviors of this multimodal courtship ritual can lead to a reproductive isolation barrier as a scaffold for incipient speciation.

Throughout the courtship process, females assess male quality [see Laturney and Billeter (2014) for review]. For example, *D. melanogaster* females tend to prefer larger males (Markow and O’Grady 2006). Also, in *D. melanogaster*, females appear to prefer “successful” males: a male is more likely to be selected as a mate by a female who observes him mating with another female (Mery *et al.* 2009). Communication between flies about male quality has been suggested (Danchin *et al.* 2010). As a result of this assessment process, females are either receptive to mate, or they decamp and leave the courtship arena. Females can reject males by flying away or, particularly if the female has mated previously, by kicking males away or extruding the ovipositor to block the male’s access (Connolly and Cook 1973). Males persist in attempting to mate but learn from the experience: *D. melanogaster* male virgins who have been repeatedly rejected become less likely to mate (Siegel and Hall 1979).

Courtship behaviors in *D. melanogaster* [reviewed in Sokolowski (2001); Figure 1] contain all the major components of courtship seen in most other species in the genus. While courtship and mating is a continual process, and difficult to divide into discrete units, we can distinguish three broad categories of this composite behavior: mate location, display, and copulation. All species in the genus *Drosophila* perform these three actions during the courtship process, although the relative order, duration, and importance of each vary between species.

### Mate location

*D. melanogaster*, and most other members of the genus *Drosophila*, encounter mates in close proximity to feeding resources. Therefore, mate location in most species is tied to the long-distance volatile plumes produced by microbes and associated decomposing substrates (Grosjean *et al.* 2011; Becher *et al.* 2012). This eliminates the need for species-specific volatile sex pheromones to broadcast mate location. Instead, a suite of compounds, many of which are components of decomposing host plants, evokes strong responses in specific olfactory sensory neurons (Laissue and Vosshall 2008), leading to aggregation or mate finding. This behavior is described further in the *Oviposition* section.

**Figure 2:**
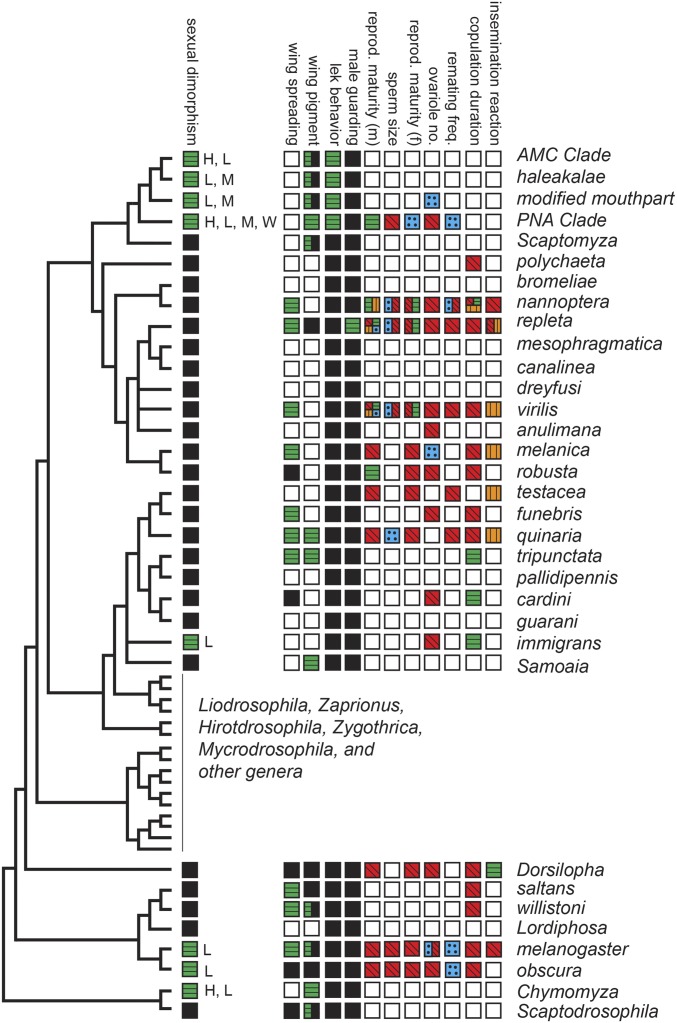
Phylogenetic distribution of reproductive behaviors and associated morphologies in species groups of *Drosophila*. All terminal taxa represent species groups [for definitions see Markow and O’Grady (2005) and O’Grady and DeSalle (2018)]. AMC indicates the antopocerus-modified tarsus clade; PNA indicates the picture wing-nudidrosophila-ateledrosophila clade. Open squares denote missing information. Characters that are polymorphic within a group show multiple colors. Sexual dimorphism (present, green with horizontal line; absent, black). Dimorphic characters, observed in males, include wings (W), forelegs (L), mouthparts (M), and head broadening (H). Wing spreading (present, green with horizontal line; absent, black). Wing pigment (present, green with horizontal line; absent, black; polymorphic, both). Lek behavior (present, green with horizontal line; absent, black). Male guarding (present, green with horizontal line; absent, black). Reproductive maturity in males and females (0–5 days, red with diagonal line; 6–10 days, green with horizontal line; 11–15 days, orange with vertical line, > 15 days, blue with dot). Sperm size [< 6 mm (short), red with diagonal line; > 6 mm (giant), blue with dot]. Ovariole numbers (< 25, red with diagonal; > 25, blue with dot). Female remating frequency (frequent, red with diagonal line; infrequent, blue with dot). Copulation duration (< 20 min, red with diagonal line; 20–60 min, green with horizontal line; > 60 min, orange with vertical line). Insemination reaction (none, red with diagonal line; moderate, green with horizontal line; strong, orange with vertical line).

While most *Drosophila* species locate mates using food-related cues, with both courtship display and copulation occurring at the feeding site, many endemic Hawaiian *Drosophila* utilize a location separate from the feeding and oviposition substrate for courtship and copulation (Spieth 1966, 1984; Figure 2). Males aggregate to engage in competitive displays to entice visiting females that are surveying prospective mating partners. This “lek behavior” is unique to Hawaiian *Drosophila*. Lek behavior correlates with a high degree of male–male aggression, with competitions between males lasting > 20 min (Spieth 1984). Within some rainforest habitats, individual trees can serve as arenas for multiple Hawaiian *Drosophila* species, creating a spatially partitioned multispecies lek (Bell and Kipp 1994).

### “Orienting” using visual and tactile displays

Once a mate is located, the first step in a successful mating is for males and females to be properly positioned so they can mate [reviewed in Sokolowski (2001); Figure 1]. This is generally referred to as orienting, where individuals of both sexes undergo a complex series of behaviors across visual, auditory, chemical, and tactile modalities to position themselves in preparation for mating. Some of these are stereotypical species-specific behaviors, while others are situational and do not necessarily occur in all mating events. Canalized, repeated display elements are considered part of the mating ritual while other, more opportunistic behaviors can be considered as a prelude to mating itself. *D. melanogaster* males will often chase females in their initial approach, eventually ending up in front of the female or slightly to her side [reviewed in Sokolowski (2001)]. This is primarily a visual display but can also include tactile aspects in which males use their forelegs to tap females either on their heads or forelegs. Both of these areas of the female contain high concentrations of chemosensory cells, which may sense pheromonal cues (Miyamoto and Amrein 2008). Thus, this behavior by males allows females to smell or taste potential mates (discussed below). Females, in turn, assess and respond to the male, either by being receptive to the mating display or by decamping and leaving the mating area.

While both sexes may be involved in signaling during reproduction, males are the primary signalers. Wings are important in orientation and males will often hold them out to their sides [reviewed in Sokolowski (2001)], perpendicular to the plane of the body, thus enlarging their visual footprint. Depending on the species, wings can range from completely clear (“hyaline”; *e.g.*, *D. melanogaster*) to lightly shaded (“infuscated”) near the cross veins, to possessing distinct apical spots or having more extensive patterns of spots, stripes, and/or darkened areas of the wings (*e.g.*, *D. biarmipes*) (Markow and O’Grady 2006; Figure 2). Males with patterned wings often wave them during visual displays to females.

Wing patterning can play an important role in species recognition. It has been suggested that it provides visual stimulation by accentuating the visibility of wing vibrations during courtship (Shevtsova *et al.* 2011) and, thus, may have gained an evolutionary advantage and spread to fixation in some species in which they have appeared (Fuyama 1979; Hegde *et al.* 2005). Indeed, many *Drosophila* species, including the agricultural pest *D. suzukii*, have independently evolved wing pigmentation spots in some males (Figure 2; True *et al.* 1999; Gompel *et al.* 2005; Prud’homme *et al.* 2006; Edwards *et al.* 2007; Werner 2015), and experiments with *D. biarmipes* showed that males with wing spots mate faster and have greater mating success than males without wing spots (Hegde *et al.* 2005). However, Roy and Gleason (2019) reported that noninvasive elimination of the wing spot of species of the *suzukii* group—*D. biarmipes*, *D. suzukii*, and *D. subpulchrella*—did not affect mating success. As noted above, different mechanisms have been used in different lineages to result in wing spots. *D. biarmipes* has acquired a transcription factor-binding site for the Engrailed transcriptional regulator through modification of a *cis*-regulatory element at the *yellow* locus (Gompel *et al.* 2005; Prud’homme *et al.* 2006). This results in the appearance of a male-specific pigmented spot on the wing. Multiple wing spots are present on the wings of *D. guttifera* as a consequence of a different regulatory mechanism that results in Wingless-mediated regulation of pigment deposition (Werner *et al.* 2010).

Visual displays are part of the mating behaviors of most *Drosophila* species. These displays include a variety of behaviors often referred to as mating “dances,” and can include wings, forelegs, and mouth parts (Markow and O’Grady 2008). Mating dances are species-specific, and distinct from the situational movements used to optimize the positions of females and males for mating. Some species, such as *D. affinis*, are so reliant on visual displays that courtship is abolished in darkness (McRobert and Tompkins 1987). One endemic Hawaiian species, *D. clavisetae*, has a particularly unique display involving both visual and chemosensory cues. Males of this species possess elongated setae on the tips of their abdomens, extending anteriorly from the cerci. These setae are used as part of the mating display: when the abdomen is everted over the top of the male’s head, the setae are extended, and a droplet of fluid is secreted and displayed in front of the female. The female then touches this droplet, obtaining taste and/or smell signals (Spieth 1966, 1974).

Another lineage of Hawaiian species, the *antopocerus* group, has elongated antennae that are densely packed with chemosensory setae on the dorsal surface and devoid of setae on the ventral surface. These antennae, along with modified setae on the male forelegs, are displayed to females during courtship. Spieth (1968) described the unique “lunge” behavior performed by this group. The elongated first and second antennal segments of the male forcibly spread the female’s wings, with the ventral surface of the third antennal segment sliding along the posterior surface of the female wing. These antennal modifications correlate with a dimorphism in olfactory centers of the brain, suggesting a role in host or mate location mediated by chemosensation, in addition to mediating the physical interaction during courtship (Kondoh *et al.* 2003).

### Courtship song

Another important mating cue is the mating “song,” usually produced by the male. *Drosophila* mating “songs” are composed of a combination of sounds generated by vibrating or “clacking” the wings, and/or by drumming the forelegs on the substrate, rather than being a true vocalization (Fabre *et al.* 2012; Mazzoni *et al.* 2013). Different *Drosophila* species have evolved distinct courtship songs, and natural genetic variation in courtship song within species bears testimony to the plasticity and evolvability of this courtship component (Gleason 2005; Arthur *et al.* 2013; Ding *et al.* 2016). Males of most species sing to females, sometimes with as many as four distinct songs (Markow and O’Grady 2006).

*D. melanogaster* creates its courtship song through wing vibrations, which give rise to distinctive pulse song and sine song components with characteristic rhythms, frequencies, and interpulse intervals (Kyriacou and Hall 1980). While the sine song is a regularly fluctuating song, the pulse song reflects irregular bursts of sound waves. These differences are the result of how the songs are produced and have a functional role in courtship (Kyriacou and Hall 1982; Ritchie *et al.* 1999; Kowalski *et al.* 2004; Talyn and Dowse 2004; Tomaru *et al.* 2004). Kyriacou and Hall (1982) showed that *D. melanogaster* females and females of its closely related species *D. simulans* mate most readily when exposed to their species-specific songs, with their characteristic interpulse intervals and oscillations. Ritchie *et al.* (1999) confirmed that *D. melanogaster* females mate most quickly when stimulated by songs typical of their own species rather than songs of *D. simulans* or *D. sechellia*. Talyn and Dowse (2004) showed that the pulse song rather than the sine song stimulates female mating. Recently, Clemens *et al.* (2018) reported a second pulse song in *D. melanogaster*. They also found that visual feedback from females influenced male song choice and that male song selection had an impact on female response (*e.g.*, receptivity *vs.* rejection). This male display also includes a complex visual component, with males ranging in position from directly in front of females to perpendicular to the female and vibrating the wings to produce the song [reviewed in Sokolowski (2001)]. The visual component continues as males position themselves directly behind the female.

The rhythmicity of the song appears to be regulated by genes associated with the regulation of circadian activity [see Dubowy and Sehgal (2017) for a review of circadian rhythms in *Drosophila*]. The *per* gene was one of the first loci implicated, based on analysis of mutants, in affecting the rhythmicity of the courtship song (Kyriacou and Hall 1980). In addition to circadian rhythmicity genes, genes identified in studies on other traits have been correlated with courtship song characteristics. For example, a study of 27 natural lines in Italy identified five polymorphisms at the *cacophony* (*cac*) locus that appeared to segregate under neutral selection and were associated with variation in interpulse interval, pulse amplitude, and cycles per pulse (Peixoto and Hall 1998; Peixoto *et al.* 2000). Finally, QTL mapping studies of courtship song qualities demonstrated that the song is a highly polygenic trait (Gleason 2005). QTL mapping studies on recombinant inbred lines of *D. melanogaster* showed variance that exceeded the variance observed in the parental lines, indicative of epistasis (Gleason *et al.* 2002). However, QTL studies have only rarely (*e.g.*, Ding *et al.* 2016) identified causal polymorphisms associated with variation in courtship song parameters.

Song production requires a neural circuit that enables manifestation of the courtship song; this circuit likely evolves with the song. Male-specific neuronal differentiation of the song circuit has been attributed to genes that comprise the sex-determination pathway. Neurons in the brain that express *fruitless*, designated P1 and pIP10 neurons, initiate the onset of courtship song [for reviews on the *fruitless*-expressing neuronal circuit that controls courtship behavior, see Yamamoto and Koganezawa (2013) and Yamamoto *et al.* (2014)]. The descending pIP10 neuron drives activity of thoracic motor neurons to shape the courtship song (von Philipsborn *et al.* 2011). In addition, regulation of sexual differentiation by *doublesex* results in male-specific expansion of dendritic arborizations of thoracic interneurons (TN1A neurons), which drive activity of the hg1 wing motor neuron (Shirangi *et al.* 2016). Studies using noninvasive imaging have identified at least seven motor neurons for the generation of courtship song that are distinct from motor neurons used to power flight (O’Sullivan *et al.* 2018).

Like many reproductive traits, mating songs evolve rapidly. Examples of rapid mating song evolution are seen within the *melanogaster* species group, where females distinguish and prefer the song from their species over that of other, even closely related, species. Moreover, males of most species in this group have two distinct courtship songs. *D. yakuba* generates two types of pulse song, one generated by wing vibration as in *D. melanogaster* and the other, termed “clack song,” which results from clapping both wings together behind the back (Demetriades *et al.* 1999). Inhibition of the descending pIP10 neurons by tetanus toxin results in loss of the clack component of the song, and optogenetic activation of the pIP10 neuron results in the production of clack song. Production of the clack song is dependent on the level of light intensity (Ding *et al.* 2019). In *D. yakuba*, under low light only clacks are produced, but under high light intensity, clacks followed by a pulse song are observed, indicating that pIP10 neurons can access both pulse and clack song circuits in an environment-dependent manner. Whereas electrophysiological properties of the pIP10 neurons are conserved between *D. yakuba* and *D. melanogaster*, a neuroanatomical comparison between these species showed significant quantifiable differences in the song circuit, with about a twofold increase in the mesothoracic triangle of *D. yakuba* compared to *D. melanogaster* (Ding *et al.* 2019). The mesothoracic triangle is a triangular-shaped structure between the dorsal pro- and mesothoracic ganglia in the ventral nerve cord, which represents a neural circuit associated with courtship behavior (Yu *et al.* 2010). An elegant study comparing the courtship songs of the closely related species *D. mauritiana* and *D. simulans* combined a classical QTL mapping approach with CRISPR technology to identify a transposable element insertion in the *slowpoke* (*slo*) gene that accounts for differences in the sine song between these two species. This gene encodes a calcium-activated potassium channel that is expressed in neurons and muscles; molecular variation at this locus may affect muscle movements necessary to generate the courtship song (Ding *et al.* 2016).

Males of species in the *repleta* group produce either one or two songs. This shows a phylogenetic distribution: taxa with one song are in one clade and those with two are in another [reviewed in Markow and O’Grady (2005)]. The range of variation is wider in the *obscura* species group, where species have either one or two songs (Markow and O’Grady 2005). In yet another variation, *D. subobscura* has entirely lost an auditory display; it does not sing during courtship. Song characteristics in the *willistoni* species group are even more variable. While *D. nebulosa* does not sing, *D. paulistorum* has two distinct songs (Ritchie and Gleason 1995; Gleason and Ritchie 1998). Two other species, *D. insularis* and *D. willistoni*, utilize three songs during courtship. Interestingly, two other species in the species group, *D. tropicalis* and *D. equinoxialis*, have independently evolved a fourth song. The *virilis* species group also shows a pattern of independent gains of courtship song (Ritchie and Gleason 1995; Gleason and Ritchie 1998). The ancestral condition is a single song, with four unrelated species each having independently evolved a second song [reviewed in Markow and O’Grady (2005)]. The high degree of variability in auditory display is likely a reflection of how important these behaviors are, not only for females to assess male quality, but also to identify conspecific males. However, songs are constrained against being too variable. Any male with a song outside the tolerances of a conspecific female may not have the opportunity to mate (Ritchie and Kyriacou 1994; Ritchie and Gleason 1995; Klappert *et al.* 2007).

Evolution of courtship song requires coevolution of the production of the song by the male and receptivity to that particular song by the targeted female. Whereas most studies have focused on the production of courtship song by the male, fewer studies have focused on the female’s perception of the song. Sound is perceived by mechanosensory antennal neurons in Johnston’s organ, which project to the antennal mechanosensory and motor center (AMMC), the first synaptic relay in the brain. This relay filters incoming information to preserve the spacing of song pulses (Tootoonian *et al.* 2012). Recordings from central neurons that innervate the AMCC of *D. melanogaster* and *D. simulans* did not show differences in responses to their pulse songs, despite the fact that each species has evolved a unique song (Tootoonian *et al.* 2012), and behavioral studies show that each species responds specifically to its species-specific song (Kyriacou and Hall 1982; Ritchie *et al.* 1999). Thus, details of how the spectral characteristics of the courtship song are represented in the female brain and the neural basis of their species divergence that results in species-specific female behaviors in response to male songs remain to be elucidated.

Evolution of courtship song may be driven by female preference (Hoikkala *et al.* 1998; Yukilevich *et al.* 2016). Females of *D. montana* use courtship song to evaluate the genetic quality of the courting male and they prefer a courtship song with short sound pulses at high frequency (Hoikkala *et al.* 1998). Therefore, males with longer sound pulses would mate less frequently and, as a consequence, songs with such pulses would become less common in the population.

Variation in courtship song can lead to sexual isolation. This is especially evident in the striking diversity of courtship songs among the > 500 species of *Drosophila* that have evolved on the Hawaiian Islands. Some species have acquired aspects of their courtship songs that are reminiscent of the complex pulse rhythm observed in crickets (*D. cyrtoloma*) or the high carrier frequency seen in cicadas (*D. fasciculisetae*). Others, like *D. silvestris*, have songs more similar to the courtship song of *D. melanogaster*, but use abdominal rather than wing vibrations to generate the song (Hoy *et al.* 1988).

Females of some species, such as members of the *virilis* species group, respond to males with a song of their own, indicating receptivity to courtship (Satokangas *et al.* 1994; LaRue *et al.* 2015). While little is currently known about why female songs evolved in this group, several lines of evidence suggest that it might be a way to recognize conspecific individuals and, in closely related species, to provide a barrier to interspecific matings. Male responses to female songs in *virilis* species range from singing and the licking of female terminalia to completely stopping courtship. Interestingly, male genitalia in the *virilis* group show little variation, suggesting that morphology is most likely not the way in which conspecific individuals recognize one another, and that premating barriers are weak or lacking in this group. Evidence from forced interspecific matings in the laboratory shows that fertile hybrids are obtained between almost all species pairs (Throckmorton 1982). This suggests that there are few postmating barriers in this group. Thus, female songs in the *virilis* group (Satokangas *et al.* 1994) may have arisen to prevent interspecific matings in a morphologically homogeneous group of genetically closely related taxa.

### Evolution of pheromone-mediated courtship signals

In addition to male wing displays, courtship and mating in *Drosophila* species are guided by chemical signals exchanged between the male and female [reviewed in Venard and Jallon (1980), Jallon (1984), Ferveur (1997, 2005), and Billeter and Wolfner (2018)].

#### Cuticular hydrocarbons:

Cuticular hydrocarbons protect against desiccation, and some components of the cuticle have been coopted as contact pheromones (Chung and Carroll 2015). Evolution of pheromonal communication requires coevolution of the production and perception of the pheromonal profiles of the interacting partners, and shifts in chemical communication, like changes in the courtship song, can establish reproductive barriers. Thus, evolution of differences in the hydrocarbon profile and response could lead to reproductive isolation. Male mate choice based on discrimination of female cuticular hydrocarbons has been implicated as the driving source for reproductive isolation between *D. simulans* and *D. sechellia* (Shahandeh *et al.* 2018). *D. melanogaster*, *D. simulans*, and *D. sechellia* evolved different sensitivities, and behavioral responses, to the aggregation pheromones (*Z*)-5-tetradecenoic acid and (*Z*)-7-tetradecenoic acid (Mast *et al.* 2014). Responses to these compounds are mediated by neurons that express *ppk23* and *ppk29* (Mast *et al.* 2014; Seeholzer *et al.* 2018). In adults, ion channels of the *pickpocket* family are expressed in *fruitless* (*fru*)-expressing neurons on the legs that respond to pheromones by mediating inhibition of courtship between males, while promoting male–female interactions (Thistle *et al.* 2012; Toda *et al.* 2012). In addition to *ppk23*, *ppk25* also promotes conspecific courtship in *D. simulans* (Ahmed *et al.* 2019).

The cuticular hydrocarbon profile is sexually dimorphic in *D. melanogaster* (Antony and Jallon 1982; Jallon and David 1987; Grillet *et al.* 2006; Dembeck *et al.* 2015) and other *Drosophila* species, including *D. sechellia* (Jallon and David 1987; Gleason *et al.* 2009) and *D. erecta* (Jallon and David 1987), but sexual dimorphism in hydrocarbon profile has not been observed in their closely related sister species *D. simulans* (Gleason *et al.* 2009). *D. melanogaster* males produce 7-tricosene as their principal cuticular pheromone, whereas females produce 7,11-dienes, such as 7,11-heptacosadiene. Analysis of DGRP strains revealed extensive variation in cuticular hydrocarbon profiles (Dembeck *et al.* 2015). Variation in hydrocarbon profiles is also evident among species of the genus *Drosophila*. Among the three closely related sister species *D. simulans*, *D. mauritiana*, and *D. sechellia*, the predominant male hydrocarbon in *D. sechellia* is 6-tricosene (Coyne 1996a). Females of *D. simulans* predominantly produce 7-tricosene, the pheromone characteristic of *D. melanogaster* males (Coyne 1996b). The predominant compound of the cuticular hydrocarbon profile of *D. sechellia* females is 7,11-heptacosadiene, whereas 7-tricosene is prevalent in the cuticular hydrocarbon profile of females of *D. mauritiana* (Coyne and Charlesworth 1997). Thus, distinct cuticular hydrocarbon compositions have evolved among closely related species within the *melanogaster* group.

*D. melanogaster* males are stimulated by the 7,11-heptacosadiene produced by the females, but this same pheromone inhibits courtship in males of its sister species *D. simulans* (Coyne *et al.* 1994; Marcillac *et al.* 2005b; Seeholzer *et al.* 2018). Evolutionary reorganization of the neural circuit that regulates the activity of P1 neurons, which is implicated in mediating courtship song (von Philipsborn *et al.* 2011), is responsible for the switch in the activation and suppression of courtship in response to 7,11-heptacosadiene between *D. melanogaster* and *D. simulans* (Seeholzer *et al.* 2018).

QTL mapping studies on hybrids derived from closely related species, such as *D. melanogaster* and *D. simulans*, or *D. mauritiana* and *D. sechellia*, have implicated loci on the third chromosome associated with generating differences in cuticular hydrocarbon composition among species (Coyne *et al.* 1994; Coyne 1996a,b; Coyne and Charlesworth 1997; Gleason *et al.* 2005) and sensing different pheromonal signatures (McMahon *et al.* 2002). Subsequent studies associated members of the desaturase gene family with sex-specific expression and the evolution of differences in cuticular pheromones between *Drosophila* species (Marcillac *et al.* 2005a; Legendre *et al.* 2008; Bousquet *et al.* 2009; Shirangi *et al.* 2009; Keays *et al.* 2011; Pardy *et al.* 2019).

The desaturase gene family, which is located on the third chromosome in *D. melanogaster*, has evolved through repeated gene duplications and diversification, with evidence of purifying selection of daughter genes (Keays *et al.* 2011). A transposon in the *Desat1* gene of *D. melanogaster* reduced the production of unsaturated hydrocarbons in both sexes, and mutant males could not discriminate sex pheromones of control flies and vice versa (Marcillac *et al.* 2005a). Expression of *DesatF* has been correlated with the production of long-chain dienes across the *Drosophila* phylogeny. Evolutionary analyses have identified multiple independent inactivations and changes in sex-specificity of this gene. These studies also identified the acquisition or loss of a functional binding site for the sex-determining transcriptional regulator Doublesex in evolutionary transitions between the acquisition and loss of sexual dimorphism of gene expression of *DesatF* (Shirangi *et al.* 2009).

African *D. melanogaster* show different cuticular hydrocarbon profiles from those of the cosmopolitan *D. melanogaster* strains that are typically used in laboratories. African *D. melanogaster* females produce high levels of 5,9-dienes and low levels of 7,11-heptacosadiene relative to the levels produced by their cosmopolitan counterparts (Ferveur *et al.* 1996; Legendre *et al.* 2008). Variation at the *Desat2* locus has been implicated in this difference (Dallerac *et al.* 2000), specifically a 16-bp deletion in the 5′ regulatory region of *Desat2* in African *D. melanogaster* relative to cosmopolitan strains (Takahashi *et al.* 2001). An elegant gene replacement study of *Desat2* alleles between a Zimbabwe population and cosmopolitan *D. melanogaster* showed that the cosmopolitan *Desat2* allele also contributed to cold resistance and starvation resistance, suggesting a connection between ecological adaptation and reproductive isolation (Greenberg *et al.* 2003). A similar demonstration connecting pheromone-producing enzymes with ecological adaptation and reproductive isolation comes from a temperature selection study of a *D. melanogaster* population from the Comoro islands. There, a few generations of selection at higher temperature resulted in an increase of 7-pentacosene at the expense of 7-tricosene and concomitant increased resistance to desiccation, and partial sexual isolation between selected and nonselected strains (Bontonou *et al.* 2013). Significant species-specific differences in cuticular hydrocarbon composition have also been documented for the closely related sibling species of the desert-dwelling cactophilic *D. repleta* group, *D. mojavensis*, *D. arizonae*, and *D. navojoa*. It has been suggested that such differences occur early in the evolution of new species (Etges and Jackson 2001).

A comprehensive survey of cuticular hydrocarbon variation in the DGRP showed that 17 DGRP lines contain the functional *Desat2* allele thought to be unique to African and Caribbean *D. melanogaster* females, and accordingly produce a high 5,9-heptacosadiene to 7,11-heptacosadiene ratio. Thus, this *Desat2* allele is also segregating among cosmopolitan *D. melanogaster* populations (Dembeck *et al.* 2015). Furthermore, genome-wide association analyses with the DGRP identified 305 and 173 genes in females and males, respectively, associated with variation in cuticular hydrocarbon profiles, of which 24 candidate genes, associated with fatty acid metabolism, were causally validated (Dembeck *et al.* 2015). Thus, variation in cuticular hydrocarbon composition is highly polygenic and is mediated by variation in the activities of enzymes that control cuticular hydrocarbon biosynthesis (Yew and Chung 2015). Variation in pheromonal communication can result from sexually dimorphic variation in the activity of desaturases that form double bonds (Labeur *et al.* 2002; Houot *et al.* 2010) and elongases that extend hydrocarbon chains (Chertemps *et al.* 2007; Ng *et al.* 2015).

Variation in pheromone-mediated chemical communication is in part due to the ratio of female-characteristic *vs.* male-characteristic cuticular hydrocarbons. Mutations in *Darkener of Apricot* (*Doa*) interfere with sex-specific splicing of *doublesex* pre-mRNA in *D. melanogaster*, resulting in the masculinization and feminization of female and male cuticular hydrocarbon profiles, respectively, along with disruption of associated courtship behavior (Fumey and Wicker-Thomas 2017).

Cuticular hydrocarbon profiles of *D. melanogaster* show extensive environmental plasticity (Rajpurohit *et al.* 2017). The composition of cuticular hydrocarbons can be modulated by time of day and social environment in *D. serrata* (Chenoweth *et al.* 2010; Gershman *et al.* 2014) and *D. melanogaster* (Kent *et al.* 2008; Krupp *et al.* 2008). Expression of cuticular hydrocarbons in males of the Australian species *D. serrata* increased when more females than males were present and resulted in greater male mating success (Gershman and Rundle 2017). Transcription of *Desat1* in oenocytes, which are the pheromone-producing cells, is under circadian control, leading to fluctuations in the accumulation of cuticular hydrocarbons over the course of a day (Krupp *et al.* 2008). Cuticular hydrocarbon profiles are also modified as a result of mating and aging (Everaerts *et al.* 2010). This may involve the epigenetic modulation of gene expression, since aging and mating are accompanied by extensive changes in histone modifications (Zhou *et al.* 2014). Thus, environmental plasticity, genotype-by-environment interactions, and plasticity in the epigenetic landscape of the genome provide a rich palette for natural selection and the evolution of behavior.

#### *cis*-vaccenyl acetate:

The pheromone 11-*cis*-vaccenyl acetate (cVA) regulates courtship behavior and intermale aggression, and attracts females to oviposition sites (Ha and Smith 2006; Kurtovic *et al.* 2007; Wang and Anderson 2010). It is made in the male’s ejaculatory bulb and transferred to females during mating, where it is found in the mating plug that forms within the bursa (uterus) of the female and decreases her attractiveness. However, this inhibitory effect on attractiveness also requires transfer to the female of cuticular hydrocarbons (rubbed off from her mate during mating), particularly 7-tricosene, made by the male’s oenocytes. These two hydrocarbons are most potent as a blend: a female with reproductive tract cVA and cuticular 7-tricosene is particularly unattractive to males (Laturney and Billeter 2016). A few hours postmating, females eject their mating plugs and with it the cVA that they received from their mates. This changes their hydrocarbon blend, making them somewhat more attractive to males, as compared to immediately after mating when they have both 7-tricosene and cVA.

Odorant-binding proteins (OBPs) are a diverse group of small proteins, some of which are thought to bind hydrophobic odorants and present them to their membrane-bound chemoreceptors (Sun *et al.* 2018). The LUSH OBP, which was originally identified as a carrier for short-chain alcohols (Kim *et al.* 1998), serves as a transporter of cVA to the OR67d receptor, mediating courtship behavior and intermale aggression (Ha and Smith 2006; Kurtovic *et al.* 2007; Wang and Anderson 2010). The OR67d receptor is expressed in trichoid sensillae of the T1 class (van der Goes van Naters and Carlson 2007) and activation of the T1 circuitry promotes courtship behaviors (Ronderos and Smith 2010). However, cVA also interacts with the OR65a receptor suppressing intermale aggressive behavior (Liu *et al.* 2011) and inhibiting courtship (Lebreton *et al.* 2014). Thus, it appears that multiple neural circuits activated by cVA regulate intermale aggression and courtship through balanced integration between the activities of both neural projections, which could conceivably be modulated by environmental factors. OBP69a has also been implicated in mediating effects of cVA on social behavior (Bentzur *et al.* 2018).

Dekker *et al.* (2015) showed that the agricultural pest species *D. suzukii* does not produce cVA, and in this species the T1 neural projection has regressed. However, *D. suzukii* has an intact OR67d receptor, and application of cVA to *D. suzukii* males reduced mating (Dekker *et al.* 2015). In the absence of an intact T1 neural projection it is possible that this effect is mediated via the OR65a receptor.

Elimination of a functional microRNA, miR-124, which influences the sex-determination pathway in *D. melanogaster* by limiting expression of the female-specific form of *transformer*, resulted in a change in pheromone profile in males. These males produced less cVA and more pentacosenes, which are attractive to males (Siwicki *et al.* 2005). This altered pheromone composition promoted male–male courtship with a concomitant reduction in male reproductive success (Weng *et al.* 2013).

## Variations in Premating and Mating Behaviors Across the Genus *Drosophila*

Near the end of the courtship ritual, *D. melanogaster* males lick the female’s genitalia with their mouthparts, prior to curling their abdomens and initiating copulation (Sokolowski 2001). Close interactions like tapping and licking may facilitate the use of cuticular hydrocarbons, or short peptides, for species recognition and mate assessment. To attempt copulation, males curl their abdomens to engage their genitalia with the female [reviewed in Sokolowski (2001)], positioning and holding females in place using a combination of “sex combs” on their forelegs and the clasper teeth (prensisetae) of the male’s genital arch; these teeth interdigitate with “vaginal teeth,” a series of bristles (setae) in the female’s external genital structures (Mattei *et al.* 2015). Males of *melanogaster*- and *obscura*-group species have sex combs, modified heavy bristles on their forelegs, that are used to position and hold females in place [reviewed in Sokolowski (2001)]. The degree of sexual dimorphism of anatomical features associated with courtship and mating varies across the genus (Markow and O’Grady 2006), from an absence of dimorphisms to relatively modest modifications like sex combs on the forelegs of the *melanogaster* and *obscura* species groups, to a wide array of wing, foreleg, and mouthpart structures observed in individual species of Hawaiian *Drosophila* (Figure 2).

Experimental removal of setae associated with male genitalia in members of the *melanogaster* group (*D. bipectinata* and *D. ananassae*) decreased the chance of copulation (Polak and Rashed 2010). Relatives in the *melanogaster* species group possess additional secondary claspers associated with the anal plate, or cerci. These “posterior lobes” are also used during positioning, interdigitating with the female’s tergites to help hold the mating pair in the appropriate position (Masly *et al.* 2011).

Despite the varied and intricate premating behaviors displayed by many drosophilids, some lineages lack evident courtship displays. For example, many males of species in the genus *Scaptomyza* do not display to females. Instead, courtship is brief, with males simply attempting to mount any females they encounter (Spieth 1966). While many *Scaptomyza* species lack secondary sexual dimorphism in wings, forelegs, or other structures, their males possess highly complex male genitalia. These genitalia often have additional lobes, setae, or other grasping structures, which help position and hold females in place. This investment in “locking” male genitalia may have allowed, or compensated for, a reduction in behaviors and morphologies related to premating displays.

Various aspects of mating, including copulation duration and remating frequency, are subject to selection and show a wide range of variation across *Drosophila*. Mating itself is a risky behavior, opening both participants to higher incidences of parasitism and predation. Longer copulations or more frequent mating would increase this risk.

Remating frequency is also highly variable across the genus *Drosophila* and can be an important factor in reproductive behaviors (Markow 2002). *D. melanogaster* females can remate roughly every 22 hr in the wild (Giardina *et al.* 2017). Cactophilic *Drosophila* remate frequently, sometimes multiple times per day (Markow 2002). Species that remate frequently have a higher incidence of sperm competition and greater variation in sperm storage structures than species that remate less frequently (Pitnick *et al.* 1999).

## Male Postmating Behaviors

After mating, a series of behavioral changes occur in females (described below), many triggered by seminal fluid proteins [reviewed in Avila *et al.* (2011); see also Bath *et al.* (2017)], that determine the success of the first mating in terms of progeny production as well as whether the female is amenable to mating again. Finally, zygotic barriers, such as genetic incompatibilities, can further reinforce species isolation by leading to the death or sterility of heterospecific zygotes.

### Evolution of male mating advantages

It is to a male’s advantage that his mate does not copulate with other males, to protect his sperm investment in the female. If the female’s attractiveness decreases after mating, the chance of the male’s sperm being competed by a rival’s also decreases. Males can use their bodies, sperm, and/or secretions from their reproductive glands to physically or chemically block the female reproductive tract, reducing the chance that another male will mate with the female prior to fertilization and the start of oviposition.

### Mate-guarding behavior

Following copulation, males of most *Drosophila* species depart, leaving the female to feed, oviposit, or mate again. Males of two species of the cactophilic *D. repleta* species group, *D. pegasa* and *D. mainlandi*, have independently evolved a mate-guarding behavior (Wasserman *et al.* 1971). Males of these species guard females by remaining on the back of the female and riding around on her while she feeds. While genitalia are not in contact for much of this time, his presence physically blocks other males from copulation, not only allowing for time for insemination but also providing the opportunity for a second copulation by the guarding male. *D. pegasa*, for example, will ride on females for extended periods of time, often > 8 hr (Gronlund *et al.* 2002), and sometimes for as long as 14 hr. Wasserman *et al.* (1971) reported “chains” of up to four males riding on the backs of females.

### Chemical mate guarding

In some species, males can prevent their mates from remating by chemical or physical means. This is mediated by molecules in the seminal fluid that are transferred during mating. In *D. melanogaster*, transfer of seminal fluids and sperm initiates at ∼5–7 min after coupling (Lung and Wolfner 1999; Gilchrist and Partridge 2000) and continues during the 15–20-min mating. Several different forms of mate guarding can occur.

First, among the seminal secretions that are transferred are components of the mating plug, a physical plug that forms within the mated female. In *D. melanogaster* at least, many of these components come from the male’s ejaculatory bulb, but some come from his accessory glands. The mating plug has been suggested to serve several functions in *D. melanogaster*, such as possibly forming a scaffold along which sperm can move (Avila *et al.* 2015a and b). But from the perspective of this article, which concerns reproductive behaviors, its most important function is to contain cVA, thus decreasing the female’s attractiveness as described above (Laturney and Billeter 2016).

Second, seminal secretions “guard” the female by decreasing her receptivity to remating; these are described in the next section.

Finally, whereas the two examples above concern mate guarding within the male’s own species, in certain interspecies matings insemination can result in the formation of a hard plug, the “insemination reaction” in the bursa of the female. This plug is generated by a chemical reaction between contributions of males (sperm and other seminal proteins) and females (Markow and Ankney 1988). The insemination reaction blocks the reproductive tract and prevents other males, even conspecifics, from copulating with a mated female. The hard plug formed as a result of the insemination reaction is different from a mating plug that forms in the bursa of a female during normal (intraspecific) matings.

## Postmating Behavioral Changes in *Drosophila* Females: Competition Between the Sexes

Postmating behaviors have been well characterized in *D. melanogaster*. Behaviors are different between virgin and mated females. First, a mated female is less attractive to courting males. During the first hours postmating, this is due to a change in her pheromonal profile due to molecules transferred from the male, as described above. However, even after expelling the mating plug along with excess unstored sperm by grooming it off with her legs, females’ mating propensity still remains low, even though she is less unattractive in a pheromonal sense (as described above).

Rather, at this stage, a *D. melanogaster* female’s receptivity to remating has decreased. Mated females actively reject males, by kicking them off or extruding the ovipositor. These responses have been attributed to the action of a peptide made in the male *D. melanogaster*’s accessory gland and transferred in his seminal fluid (Baumann *et al.* 1975; Chapman *et al.* 2003; Liu and Kubli 2003). This 36-amino acid “sex peptide” binds to a G protein-coupled receptor called SPR (Sex Peptide Receptor; Yapici *et al.* 2008), and potentially to at least one more protein (Haussmann *et al.* 2013), to exert its action. A small number of neurons that innervate the female’s reproductive tract are sufficient for the loss of receptivity after mating (Häsemeyer *et al.* 2009; Rezával *et al.* 2012, 2014; Yang *et al.* 2009); at least some of these express SPR. During storage, the sex peptide is cleaved to release its C-terminal active region from the sperm. This released region binds to its receptor, keeping the female’s mating receptivity down. Viewed from the perspective of the previous section in terms of benefits to the male of decreasing the chance of his mate’s remating, the sex peptide provides the long-term chemical guarding once the male-derived hydrocarbon pheromones are gone.

In the absence of the sex peptide, copulation itself can decrease female receptivity through the activation of mechanosensory neurons (Shao *et al.* 2019). These neurons communicate with a pair of lateral sensory ascending neurons in the abdominal ganglion that project to neurons expressing myoinhibitory peptides in the brain. Even though sex peptide transferred from the male does not contribute to this particular aspect of the decreased female receptivity, SPR is essential for mediating the behavioral switch, suggesting that an endogenous SPR ligand is released during copulation (Shao *et al.* 2019).

Interestingly, *D. melanogaster* females normally remain less receptive to remating for 10–14 days, for as long as they contain sperm. This is because the sex peptide binds via its N-terminus to sperm, allowing it to be stored in females long-term (Peng *et al.* 2005). Indeed, this has been posited as a reason for an unusual feature of *Drosophila* sperm: they are very long in some species. Sperm size varies widely across the genus *Drosophila* (Pitnick *et al.* 1995; Gage 2012), from species with relatively short sperm of ∼1 mm in length (*e.g.*, *D. melanogaster*) to species with giant sperm that approach 6 cm in length (*D. bifurca*; Figure 2). Peng *et al.* (2005) suggest that this great length has arisen because longer sperm can hold more sex peptide, permitting longer (or better) dosing of the female. While this is an attractive hypothesis, it is not clear whether receptivity-regulating seminal proteins in all species with long sperm bind to those sperm. The potential advantage of long sperm for holding sex peptide(s) is balanced by cost: it is energetically demanding to make such long sperm, so species like *D. bifurca* with very long sperm invest in length over sperm numbers.

Several other behaviors change in *D. melanogaster* females after mating, most also due to the action of the sex peptide. Many of these can be understood in terms of increased egg production by mated females. Sex peptides cause mated females to eat more (Carvalho *et al.* 2006), which provides more resources for their egg production; their diet also changes to more protein-rich (Ribeiro and Dickson 2010; Vargas *et al.* 2010) and their guts grow and increase their absorptive capacity (Cognigni *et al.* 2011; Apger-McGlaughon and Wolfner 2013; Reiff *et al.* 2015). Receipt of the sex peptide also causes a decrease in females’ siesta sleep (Isaac *et al.* 2010), presumably giving them more opportunities to move around and lay eggs. Finally, female–female aggression increases postmating (Bath *et al.* 2017), again as a result of the action of the sex peptide. Presumably this increased aggression aims to increase a female’s access to limited food resources.

Mated females also ovulate at a higher rate, but this is regulated by a different seminal protein, ovulin (Heifetz *et al.* 2000). Ovulin is a 264-amino acid protein (Monsma and Wolfner 1988) that is also made in the male’s accessory gland and transferred to females, where it is proteolytically processed (Park and Wolfner 1995). Through an as yet unknown receptor and cellular target, ovulin causes an increase in octopaminergic signaling in the female’s reproductive tract (Rubinstein and Wolfner 2013). In turn, this relaxes the muscles that wrap the female’s oviduct, permitting the passage of an oocyte for ovulation (Rubinstein and Wolfner 2013; Mattei *et al.* 2015). Another muscle-based behavior in the reproductive tract involves the storage of sperm from the mating. *Drosophila* sperm are long, and movement to their storage sites appears to be assisted by contractions and relaxations of the bursa or uterus (Adams and Wolfner 2007), and potentially by a scaffold provided by the mating plug (Avila *et al.* 2015b) that coagulates inside the female. These uterine contractions and relaxations are triggered by the action of other seminal proteins, such as the glycoprotein Acp36DE (Avila and Wolfner 2009).

Finally, mated *D. melanogaster* females show changes in their oviposition site behaviors (Becher *et al.* 2012). Oviposition site selection includes evaluating sites for ones with low likelihoods of parasites and high likelihoods of food. Females also tend to lay eggs where other females lay; cVA ejected with the mating plug can form part of the cue to attract other females to the site of oviposition (*e.g.*, Bartelt *et al.* 1985; Lebreton *et al.* 2012; Billeter and Levine 2015). ILP7-expressing neurons are important in the selection of egg-laying sites by females (Yang *et al.* 2008). Also, females are attracted to acetic acid, and avoid UV light, when they are laying eggs. Their attraction to acetic acid is not triggered by mating *per se* but rather by the stretching of the females’ reproductive tract as eggs pass through the tract (indirectly, there can be a mating effect, as mating increases egg production and ovulation; *e.g.*, Heifetz *et al.* 2000, 2001). Some *ppk* mechanosensory neurons that innervate the lateral and common oviducts (tract-tiling *ppk* neurons) are involved in sensing that eggs are present in the reproductive tract (stretching the tract); these are different from the *ppk* neurons that are reported to mediate the response to the sex peptide. The aversion to UV appears to be mediated by bitter-sensing neurons in the female’s proboscis, via certain isoforms of the dTRPA1 channel (Guntur *et al.* 2017).

Seminal proteins delivered by the male often show evidence of rapid evolution under positive selection (*e.g.*, Haerty *et al.* 2007). The sex peptide is found in many, but not all *Drosophila* species (*D. mojavensis* and *D. grimshawi* appear to lack it) and not outside of Drosophilidae (Tsuda *et al.* 2015; Tsuda and Aigaki 2016). Its receptor, SPR, is found in most insects (Yapici *et al.* 2008), likely because of this protein’s ancestral role as a receptor for myoinhibitory peptides (Kim *et al.* 2010; Poels *et al.* 2010). The sex peptide gene occurs in more than one copy in several *Drosophila* species (Cirera and Aguadé 1997, 1998). Tsuda *et al.* (2015) showed that *D. melanogaster* sex peptide can induce postmating responses and binds to the oviduct in all *melanogaster*-group species tested, but did not induce postmating responses in females of the *obscura* or *willistoni* groups, or *D. virilis*, and injection of conspecific sex peptides did not either. This suggested to Tsuda and Aigaki (2016) that the induction of postmating responses via sex peptide/SPR interaction has uniquely evolved in the *melanogaster* group (Tsuda and Aigaki 2016). The function of the sex peptide in the other *Drosophila* species remains unknown.

Ovulin is also very rapidly evolving at the primary sequence level and is almost impossible to recognize, even in *D. pseudoobscura* (Clark *et al.* 1995). The seminal protein Acp36DE also shows evidence of rapid evolution (Wagstaff and Begun 2005). Such rapid evolution is also evident in mammalian seminal proteins (*e.g.*, Dean *et al.* 2011) and occurs against a backdrop of conserved molecular functions in seminal fluid (Mueller *et al.* 2004). For example, there are proteases and protease inhibitors in the seminal fluids of all species examined (insects and mammals), but their primary sequences can be very different [reviewed in Laflamme and Wolfner (2013)]. This rapid evolution may be driven by conflicts between the interests of individual males, and/or between the reproductive strategies of males and females. In terms of the former, sperm competition occurs in *D. melanogaster* females, which can mate three to five times in nature (Reinhart *et al.* 2015). In this case, there will be selection for molecules, including better and new ones, that can improve or enhance the chance that a male’s sperm will be stored, or will compete well against sperm of other males. In terms of the latter, the changes that are caused in mated females by seminal proteins from the male may be more beneficial from his perspective than they are from hers.

Decreasing remating can be beneficial to a male in preventing competition from rivals’ sperm, but it may be less advantageous for a “choosy” female who can benefit from the chance to select among the sperm of different males. Similarly, increasing feeding and egg production, and decreasing the female’s sleep may be beneficial to the male in terms of enhancing the fertility of the mating, but it may decrease the female’s health and longevity; indeed, mating, and the sex peptide in particular, decreases the longevity of mated *D. melanogaster* females (Chapman *et al.* 1995; Wigby and Chapman 2005). Thus, there may be selection on females to develop resistance to male seminal proteins or other modulators, causing strong selection pressure for males to evolve more potent versions of those proteins, or new ones that can be coopted for their functions. In this light, it is interesting that ovulin appears to act at the very top of the ovulation-regulatory cascade, turning up octopaminergic signaling rather than tweaking the details of octopamine’s action. It is conceivably easier to evolve a new switch to turn up an existing, conserved, physiological pathway than to modify the components of that pathway (Kirschner and Gerhart 1998; Hoke *et al.* 2019). In a somewhat analogous argument, although the sex peptide is a novel molecule, it functions as a new ligand for a conserved, previously existing receptor; SPR is originally the receptor for myoinhibitory peptides. In addition, we can hypothesize that rapid evolution of inducers of postmating behaviors may help in species isolation. In such cases, if heterospecific matings occur despite premating isolation mechanisms, having highly species-specific molecules that induce postmating responses in females may contribute to decreasing the reproductive success of the heterospecific pair.

## Oviposition

Oviposition behavior in *Drosophila* can be divided into two phases: (1) finding the oviposition site or “host plant selection,” and (2) the act of physically inserting the egg into the selected substrate (Jaenike 1985, 1990). The former requires chemosensory cues that are interpreted via the olfactory and gustatory system. The latter involves a series of characteristic movements leading up to and including the placement of the egg, and may be correlated with special morphological adaptations depending on the physical structure and stage of decomposition of the oviposition site.

### Host plant selection

With a few exceptions, most *Drosophila* species are saprophagous and rely on microbes (*i.e.*, yeasts and bacteria) to break down plant material upon which they feed. Some species, like *D. melanogaster*, are generalists that have been reared successfully from decomposing fruits, fungi, and flowers of many different plant species, as well as a range of other substrates (*e.g.*, decomposing animal matter). However, the majority of species in the genus *Drosophila* display a degree of fidelity in oviposition site selection in or on a given host plant taxon (Throckmorton 1975; Magnacca *et al.* 2008). This is likely due to the fact that flies are targeting microbes that have a characteristic niche on a specific plant or group of plants (Ort *et al.* 2012). Interestingly, microbes are not the only drivers in this system, as it is clear that plant secondary compounds also play a role in the selection of an oviposition site, either as deterrents or as attractors [reviewed in Barker and Starmer (1982)]. For example, volatile profiles of necrotic cacti species (and many other plants) are highly attractive to some *Drosophila* species. However, once *Drosophila* arrive on a substrate, they may find that toxic plant compounds make that substrate unsuitable. Therefore, when assessing a given substrate, *Drosophila* must make decisions based on a complex calculation involving microbial community profile, plant species, and stage of decay of a substrate (Yang *et al.* 2008; Guntur *et al.* 2017). Since the microbial community is linked to the host plant and decay condition, we will refer to this behavior broadly as host plant selection.

Most *Drosophila* species fit along a spectrum from oviposition generalists, where a single species of fly may use many different plants for egg laying and development, to oviposition specialists that lay eggs on only a single plant species. O’Grady and Markow (2012) recognized four broad categories when discussing oviposition preference in *Drosophila*. “Broad generalists” can utilize a wide range of resources spread across feeding guilds. *D. melanogaster* is an example of a broad generalist and will oviposit in nearly any decomposing fruit, flower, or fungus.

“Substrate generalists” feed on a single type of host plant, but can utilize a wide range of unrelated host species of similar types. Members of the *tripunctata* species group fall into this category; they oviposit on fungi but, rather than lay eggs on a single fungal species, they oviposit on and develop in many different unrelated fungal species (Throckmorton 1975).

“Substrate specialists” utilize a single substrate type from a single clade of host plant. For example, the cactophilic *repleta* species group oviposits on only a single family of plant, Cactaceae (Oliveira *et al.* 2012). However, within this single family there are many different host species, and various *repleta*-group species may be generally attracted to and utilize many. Hawaiian *Drosophila* are also categorized as substrate specialists (Magnacca *et al.* 2008) since they oviposit on a single substrate from a single plant lineage (*e.g.*, leaves of Araliaceae).

The most narrowly defined class of specialists are the “true specialists.” These species use only a single type of substrate from a single species of host plant. *D. sechellia*, which uses only the noni fruit (*Morinda citrifolia*) for oviposition, is an example of a specialist species (Jones 2005). *D. pachea*, a species that only uses senita cactus (*Pachycereus schottii*), is another (Lang *et al.* 2012).

Female ovipositor and egg morphologies often vary with preferred host sites. This has been studied extensively in the Hawaiian *Drosophila* and *Scaptomyza* (Craddock and Kambysellis 1990; Kambysellis *et al.* 1995). Species of *Scaptomyza* tend to have simple, fleshy ovipositors without many bristles or even sclerotization (the darkening of the cuticle that indicates cross-linking of proteins and structural strength). These taxa oviposit in flowers or on the surfaces of leaves, effectively “dumping” eggs, rather than inserting them into the host substrate (Kambysellis *et al.* 1995). The respiratory filaments of the eggs of these species are correspondingly very short or completely absent, reflecting the abundance of available oxygen and the relatively dry oviposition substrates. In contrast, females of the *Drosophila* *picture wing* species group possess large, strong ovipositors with many bristles (Kambysellis *et al.* 1995). These species insert their eggs deep into rotting wood or other decomposing plant tissue. Respiratory filaments in these species are elongated, so eggs can access oxygen during development. In the species just discussed, ovipositor length, oviposition depth, and respiratory filament length are highly correlated.

### Attraction to or repulsion from oviposition sites

**Figure 3:**
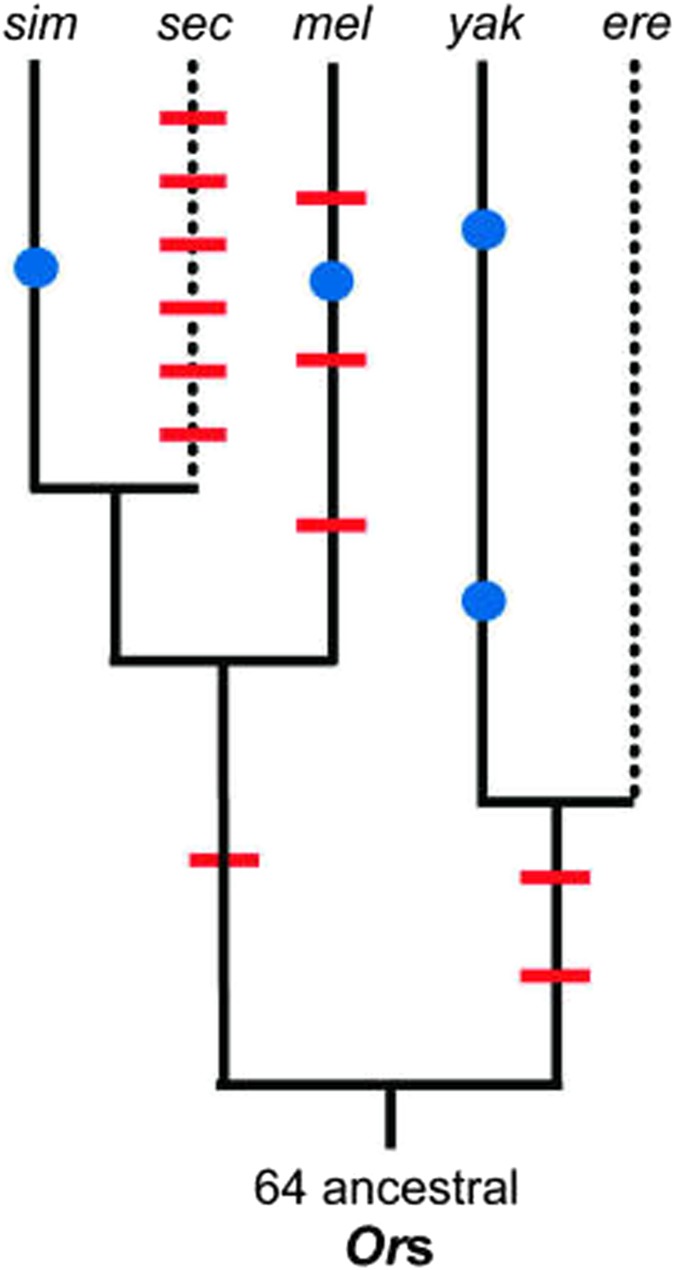
Lineage-specific gene loss and gain in the *Or* family. Diagram that illustrates gene-loss events (red slashes) and duplications (blue dots) in the *melanogaster* subgroup, including *D. simulans* (*sim*), *D. sechellia* (*sec*), *D. melanogaster* (*mel*), *D. yakuba* (*yak*), and *D. erecta* (*ere*). Generalist lineages are shown by solid black lines and specialist lineages by dotted lines. The timing of events was inferred via parsimony [modified from McBride *et al.* (2007), with permission].

Chemical recognition features prominently in how *Drosophila* select host plants for feeding and oviposition. The ability to feed on a food source that is repellent to competing species reduces competition, and ensures a reliable niche for a species that is adapted to and specialized on a specific plant. For example, the noni plant, *M. citrifolia*, produces hexanoic and octanoic acid that are repellent to most *Drosophila* species (Amlou *et al.* 1998; Jones 2005). However, *D. sechellia* is attracted to *Morinda* and, as mentioned above, can successfully oviposit and complete development on this plant. Host plant specialization of *D. sechellia* has arisen through several distinct genetic adaptations, including rapid evolutionary adaptations of its chemoreceptor repertoire (McBride 2007; McBride *et al.* 2007; Figure 3). Adaptation to *Morinda* involves expression of two *Obp* genes, *Obp57d* and *Obp57e*, which are expressed in taste sensilla on the legs. Wild-type expression of these two OBPs results in the avoidance of hexanoic and octanoic fatty acids. Conserved *cis*-regulatory elements have been identified that govern expression of these OBPs (Tomioka *et al.* 2012). Matsuo *et al.* (2007) reported that a 4-bp CCAT insertion upstream of the *Obp57e* gene in *D. sechellia* prevents its expression, while its open reading frame remains intact. However, Dworkin and Jones (2009) identified a premature stop codon in the *D. sechellia* *Obp56e* gene resulting in a loss-of-function allele. It is possible that multiple *Obp* alleles have evolved in the *D. sechellia* lineage that have the same end result on host preference behavior, namely the abolition of avoidance of hexanoic and octanoic acid. Deletion of *Obp57e* and *Obp57d* in *D. melanogaster* also resulted in altered behavioral responses to hexanoic acid and octanoic acid (Matsuo *et al.* 2007). Furthermore, studies on interspecies hybrids between *D. melanogaster Obp57d/e* knockout flies and *D. simulans* or *D. sechellia*, shifted oviposition site preferences of the hybrid offspring to that of *D. simulans* or *D. sechellia*, respectively (Matsuo *et al.* 2007).

The olfactory system of *D. melanogaster* has been well characterized. Olfaction is mediated through odorant receptors expressed by olfactory sensory neurons in sensilla on the third antennal segment. Each neuron expresses a single odorant receptor from among the chemoreceptor repertoire and axons of olfactory sensory neurons that express the same receptor converge on output neurons in the antennal lobes, forming spherical structures of neuropil: glomeruli. Combinatorial activation of olfactory sensory neurons is translated in a spatial and temporal pattern of glomerular activity that encodes the concentration and quality of the odor [reviewed by Joseph and Carlson (2015)].

Dekker *et al.* (2006) reported femtogram-level sensitivity of *D. sechellia* antennae to the host plant odorant methyl hexanoate with an approximately threefold overrepresentation of neurons responding to this odorant, compared to *D. melanogaster*, and a concomitant increase in volume of the glomerulus to which they project. Ibba *et al.* (2010) reported that the A neuron of the ab3 sensillum responds to hexanoate esters and projects to its corresponding enlarged DM2 glomerulus, whereas ab3B neurons respond to 2-heptanone, another volatile compound from the *Morinda* fruit, and also project to an enlarged glomerulus. Subsequently, Prieto-Godino *et al.* (2017) studied acid-sensing circuitry, and found that a single amino acid change in the IR75b receptor in *D. sechellia* in ac3 sensilla confers sensitivity to hexanoic acid and results in attraction to this odorant. This switch in odorant response profile is associated with concomitant expansion of the DL2d glomerulus, which receives projections from IR75b-expressing neurons. However, higher-order circuits appeared unaffected (Prieto-Godino *et al.* 2017). In addition to the roles of OBP56e, OBP57d, OBP57e, and IR75b in mediating host preference in *D. sechellia*, gene expression studies identified several other genes that showed extensive upregulation in *D. sechellia* compared to its sister species *D. simulans*, including *Or22a* (Kopp *et al.* 2008) *Obp50a*, *Or85c*, and *Ir84a* (Shiao *et al.* 2015).

*D. erecta*, a close relative of *D. melanogaster*, has evolved host specialization for screw pine fruits (*Pandanus sp*.). Similar to the situation with *D. sechellia* and *M. citrifolia* volatiles, the number of olfactory sensory neurons that respond to 3-methyl-2-butenyl acetate, a volatile produced by the *Pandanus* fruits, has increased in *D. erecta* with corresponding enlarged glomeruli in the antennal lobe. Exposure to this odorant induces oviposition in *D. erecta*, but not *D. melanogaster*, females, providing yet another example of an exquisitely tuned olfaction-mediated host plant relationship (Linz *et al.* 2013).

Another well-studied example of behavioral adaptation in *Drosophila* comes from the cactophilic species *D. mojavensis*, which feeds and oviposits on decomposing cactus in Arizona, the Mojave Desert, Baja California, the Sonoran Desert, and Catalina Island off the coast of California. Different races of *D. mojavensis* have diversified in terms of preferences for the different types of cacti endemic at each location (Newby and Etges 1998). Electrophysiological and behavioral studies have shown olfactory adaptations to volatiles from different cacti (Date *et al.* 2013), and RNA-sequencing analyses have revealed differential expression of members of the *Or* gene family between the different populations (Crowley-Gall *et al.* 2016). The geographical separation and different behavioral niches among the *D. mojavensis* populations provide a scenario for incipient speciation (Pfeiler *et al.* 2009). Indeed, analyses of genome sequences between *D. mojavensis* and its close relatives, *D. arizonae* and *D. navojoa*, showed fixed inversion differences in three of their six chromosomes (Sanchez-Flores *et al.* 2016).

From an evolutionary perspective, specialization is beneficial because it reduces interspecific competition for resources. However, specialization can also be risky if a host plant or other resource becomes scarce in the future. Understanding the genetic changes behind specialization is important because it yields insight into the mechanisms of specialization and helps in understanding how flexible a given taxon might be in the face of changing environmental factors. The studies above on *D. sechellia*, *D. erecta*, and some species of cactophilic *Drosophila* highlight some of the recent progress made on the evolution and genetics of specialization in *Drosophila*.

### Adaptation to fresh fruit

Unlike most *Drosophila* species, *D. suzukii* females are attracted to ripening fruit for oviposition. This adaptation has resulted in *D. suzukii*, often referred to as “spotted-wing *Drosophila*,” emerging as a major agricultural pest over the last decade as it has spread from its native range in Asia to Europe and North America (Walsh *et al.* 2011). Infestation of fresh fruit requires both morphological and chemical adaptations. *D. suzukii* females possess an enlarged, heavily serrated ovipositor relative to other taxa in the *melanogaster* species group. This ovipositor enables them to penetrate the skin of ripe fruit, such as strawberries, cherries, and cane fruits (Mitsui *et al.* 2006; Atallah *et al.* 2014; Cini *et al.* 2014).

Evolutionary changes in the olfactory receptor repertoire have also occurred that predispose *D. suzukii* to selecting fresh fruit as an oviposition substrate (Keesey *et al.* 2015). Comparisons of the *Or* gene repertoire of *D. suzukii* with those of its closely related species *D. biarmipes* and *D. takahashii* showed duplications of *Or23a* and *Or67a* in *D. suzukii*, with evidence for positive selection at the *Or67a* locus. In addition, *Or74a*, *Or85a*, and *Or98b* were pseudogenized in *D. suzukii* (Hickner *et al.* 2016). Furthermore, behavioral studies show that *D. suzukii* is attracted to the odor of ripe strawberry and that oviposition behavior on ripe fruit is reduced when the common odorant coreceptor gene, *Orco*, is knocked down by RNA interference or eliminated through CRISPR deletion (Karageorgi *et al.* 2017). However, it should be noted that in the DGRP, pseudogenes of chemoreceptors segregate in the population along with their intact counterparts apparently shielded from selection through functional redundancy; therefore, segregation of intact counterparts of pseudogenes in the *D. suzukii* population cannot be excluded.

### Leaf mining and herbivory

Although saprophagy is the major lifestyle of most drosophilid species, a few have adapted to novel oviposition substrates. One such adaptation is the evolution of true herbivory observed in many species of the genus *Scaptomyza*. This involves oviposition on living plant material and the subsequent development of larvae miners in the interstitial space between the top and bottom layer of leaves. The evolution of this behavior has required a suite of morphological and chemical changes.

*Scaptomyza flava* oviposits, and its larvae feed, on plants of the family Brassicaceae, *e.g.*, *Arabidopsis thaliana* (Whiteman *et al.* 2011; Goldman-Huertas *et al.* 2015). Evolutionary analysis of the odorant receptor repertoire of *S. flava* shows stepwise loss or pseudogenization of receptor genes that respond to short-chain aliphatic esters, commonly found in yeast, and duplication at the *Or67b* locus has resulted in three paralogs with evidence for positive selection (Goldman-Huertas *et al.* 2015). Interestingly, the *D. melanogaster* OR67b receptor also responds to the green-leaf volatile (*Z*)-3-hexenol (Galizia *et al.* 2010), suggesting a role for attraction to leaf volatiles in nonherbivorous *Drosophila*.

### Carnivory

While most *Drosophila* species are attracted to rotting plant and fungal matter as oviposition substrates, there are a few with more specialized and unusual ecological niches (Ashburner 1981). Several of these are in the genus *Scaptomyza*, a lineage that arose on Hawaii and has since escaped to colonize all continental landmasses except Antarctica. There are endemic Hawaiian *Scaptomyza* in the subgenus *Titanochaeta* that are predators on spider eggs sacs (Wirth 1952). The female fly oviposits in the eggs and the larvae develop in the sac by eating the spider eggs, all while being guarded by the mother spider. The larval development of *Titanochaeta* remains unknown, but the sister lineage, the subgenus *Engiscaptomyza*, larviposits rather than laying eggs (Throckmorton 1975). This is quite common in some flies, particularly those that are predators or parasitoids. Members of *Engiscaptomyza* are suspected to be predators on endemic Hawaiian land snails, an ecological niche that might require an extremely short or nonexistent egg phase. Other parasitic drosophilid species, including members of the genus *Cacoxenus*, oviposit in bee nests (Ashburner 1981). Another unusual oviposition site used by three unrelated species of *Drosophila* is the green gland of giant land crabs (Carson 1974). This has arisen in parallel on islands of the South Pacific and Caribbean. The genetic underpinnings of the evolutionary trajectories that have given rise to these diverse ecological specializations in the genus *Drosophila* remain yet to be explored.

## Perspectives

With its multiple sequenced and characterized species, multiple interspecies lines for examining intraspecies variation, and a vast array of publicly available resources, *Drosophila* offer unique opportunities for investigating the evolution of complex behaviors, such as reproductive behaviors, at both micro- and macroevolutionary scales. Much is known about the diversity of mating strategies, and ecological adaptations among members of the genus have been extensively documented. Yet, we still know little about the genetic basis of the evolutionary forces that drive these adaptations. The ability for one-way hybridization between several closely related sister species (*e.g.*, *D. melanogaster*, *D. simulans*, *D. sechellia*, and *D. mauritiana*) provides opportunities to assess the relationship between the diversification of reproductive behaviors and speciation.

Population and quantitative genetic studies that use well-annotated whole-genome data sets to identify genes and genetic networks associated with different elements of reproductive behaviors, across multiple species, may provide insights as to how these behaviors have evolved and diverged among various lineages within the genus *Drosophila*. It will be important to examine the evolution of behaviors from the contexts of trade-offs, adaptations to the social and abiotic environment, and responses to different selective pressures. Since behaviors are expressions of the nervous system, neurobiological studies are necessary to identify adaptations at the level of functional neural circuits that accompany genomic adaptations. It is here of interest to note that a study of the visual and olfactory structures among 62 *Drosophila* species showed that expansion of visual structures occurred at the expense of the size of the third antennal segment, which mediates olfaction, and vice versa (Keesey *et al.* 2019). This trade-off could be accounted for by a shared larval developmental blueprint, the eye–antennal imaginal disc, and could affect bias of sensory input for the identification of hosts and mates (Keesey *et al.* 2019).

Comparative studies within and integrating genetic/genomic, behavioral, ecological, and neurobiological approaches across multiple *Drosophila* species may provide insights into the selective forces that led to the evolution of reproductive behavior, the ultimate determinant of fitness.
